# Magnetic Field Sensors’ Calibration: Algorithms’ Overview and Comparison

**DOI:** 10.3390/s21165288

**Published:** 2021-08-05

**Authors:** Konstantinos Papafotis, Dimitris Nikitas, Paul P. Sotiriadis

**Affiliations:** Department of Electrical and Computer Engineering, National Technical University of Athens, 157 80 Athens, Greece; el15006@central.ntua.gr

**Keywords:** magnetic sensor, calibration, algorithms, review, comparison

## Abstract

The calibration of three-axis magnetic field sensors is reviewed. Seven representative algorithms for in-situ calibration of magnetic field sensors without requiring any special piece of equipment are reviewed. The algorithms are presented in a user friendly, directly applicable step-by-step form, and are compared in terms of accuracy, computational efficiency and robustness using both real sensors’ data and artificial data with known sensor’s measurement distortion.

## 1. Introduction

Magnetic field sensors (magnetometers) are nowadays widely used in a plethora of commercial, industrial, marine, aerospace and military applications. Their applications include but not limited to navigation and attitude estimation, geophysical surveys, archaeology, entertainment devices, consumer electronics and others.

In most applications, sensor’s calibration is essential in order to achieve the desirable accuracy level. The purpose of magnetic field sensors’ calibration is a twofold. First, as in the case of every measurement unit, calibration ensures that the measurement of the standalone sensor corresponds to the actual value of the magnetic field. To do so, calibration must compensate for all static (manufacturing imperfections etc.) and active (temperature, humidity, etc.) phenomena effecting the accuracy of the sensor’s measurement. In addition, when a magnetic sensor is embedded in a larger system, other components of the system may cause disturbances (both static and active ones) to the local magnetic field. Static disturbances are usually caused by magnetic and ferromagnetic materials in the vicinity of the sensor; called hard-iron distortion and soft-iron distortion respectively (more information are given in Section 2). Mechanical or electronic structures embedded in the system, such as motors and coils could also actively distort the local magnetic field and cause significant measurement error.

This review paper focuses on algorithms correcting the dominant linear time-invariant (static) measurement errors, requiring no special piece of equipment for their application. Such algorithms are most commonly used for in-situ calibration of magnetic field sensors which are usually in chip form and embedded in larger systems. The paper presents seven representative calibration algorithms for three-axis magnetometers and compares them in terms of accuracy, robustness, computational efficiency and ease of deployment. The seven algorithms are briefly presented, to introduce all required mathematical expressions, and are summarized in an easy-to-develop, step-by-step form. For the details of the algorithms, the reader is referred to the original works.

The selection of the particular algorithms was done based on their popularity and on our attempt to present as many different calibration approaches as possible. The TWOSTEP [1] algorithm is one of the first algorithms that addressed the full calibration problem (and probably the most popular one). At a later time, Elkaim and Vasconcelos [2] proposed a geometric approach of TWOSTEP which is also very popular. At the same time, Dorveaux et al. [3] offered a non-linear formulation of the problem and they treated it in an innovative, strictly iterative way. In addition, Wu and Shi [4] suggested the most complete formulation of the calibration problem as an optimal maximum likelihood estimation one. The TWOSTEP algorithm, as well as the algorithms proposed by Vasconcelos et al. and Wu et al., consist of a first step deriving an initial solution, and, a second step for improving it. On the other hand, Papafotis and Sotiriadis [5] recommended an iterative approach based on a twofold minimization, which was shown to be extremely effective. Furthermore, a real-time approach by Crassidis et al. [6] using the popular Kalman Filter is discussed. Finally, to represent the recent trends towards Machine Learning, an Artificial Intelligence (AI) method applying Particle Swarm Optimization on the estimation problem is explored [7].

Please note that this review focuses on works for in-situ calibration of three-axis magnetic field sensor without using any special piece of equipment or any other additional sensor. Thus, several interesting works dealing with magnetometer’s calibration, in combination with inertial sensors, [8,9,10,11,12] are not included in this work.

The rest of the paper is organized as follows. First, a standard error model for three-axis magnetic field sensors is presented in Section 2. In Section 3, Section 4, Section 5, Section 6, Section 7, Section 8 and Section 9, seven representative algorithms are discussed in chronological order of publication. In Section 10, a method for generating artificial data is proposed and algorithms are evaluated via extensive Monte Carlo simulation to identify their performance. In addition, the algorithms are evaluated using several real sensor’s measurements in order to evaluate their performance under real-world conditions. Finally, Section 11 summarizes our findings and provides brief comments for each algorithm. The notation used along the paper is presented in Table 1.

## 2. Magnetic Field Sensor’s Error Sources and Measurement Model

In this section, the most important linear, time-invariant error sources of three-axis magnetic field sensors are presented. Based on them, a mathematical model relating the sensor’s measurement with the actual value of the magnetic field is derived.

The total output error of a magnetic sensor is a combination of several error sources related to the sensing element itself, the instrumentation electronics, manufacturing imperfections and distortions caused by magnetic and ferromagnetic materials in the vicinity of the sensor. The linear, time-invariant error sources with the most significant contribution in the total sensor’s error, are listed below:

- **Bias, or offset**; all magnetic sensors suffer from bias, which is a constant distortion. In many cases, it is the most important defect in the sensor’s overall error. A 3×1 vector, $h_{s}$, is used to model it.
- **Scale-factor** error represents the input-output gain error of the sensor. It is modeled by a 3×3 diagonal matrix, $T_{sf}$.
- **Cross-coupling or non-orthogonality** inaccuracies are resulted by the non-ideal alignment of the sensor’s axes during manufacturing and are modeled by a 3×3 matrix, $T_{cc}$.
- **Soft-iron distortion** is caused by ferromagnetic materials in the vicinity of the sensor, attached to the sensor’s coordinate frame. Those materials do not generate their own magnetic field, but instead alter the existing magnetic field locally, resulting in a measurement discrepancy. This effect is modeled by a 3×3 matrix, $T_{si}$.
- **Hard-iron distortion** is due to magnetic materials attached to the sensor’s coordinate frame. As a consequence of the persistent magnetic field created by those materials, the sensor’s output has a constant bias. Hard-iron distortion is modeled by a 3×1 vector, $h_{hi}$.
- **Random noise** is the stochastic error in the sensor’s output. It is induced by the sensor’s mechanical and electrical architecture. It is modeled by a 3×1 vector, ε, and it is most commonly assumed to be a sequence of white noise, i.e., $\varepsilon ∼N(0,\;\sigma^{2})$.

Let *m* be the 3×1 true magnetic field vector and *y* be the 3×1 measurement vector. With the aforementioned error terms in mind, a widely accepted and well-referenced measurement model for a three-axis magnetometer is the following [1,2,4,5,6,7,13,14]

(1) $$y=T_{sf}T_{cc}\left(T_{si}m+h_{hi}\right)+h_{s}+\varepsilon$$

In most applications, the exact contribution of each error term in (1) is of no concern and, thus, instead of (1), most calibration algorithms use the following, compact form of (1)

(2) y=Tm+h+ε

where $T≜T_{sf}T_{cc}T_{si}$ and $h≜T_{sf}T_{cc}h_{hi}+h_{s}$.

This work focuses on algorithms intended to be used with magnetic field sensors requiring no special piece of equipment. In such cases, the calibration is done in the sensor’s (body) coordinate frame implying that both the measurement vector, *y* and the true magnetic field vector, *m* in (2) are expressed in the senor’s coordinate frame.

Note that when expensive laboratory equipment is not available, both the calibration parameters *T* and *h* in (2), and the magnetic field vector, *m*, are unknown. Thus, in most works, multiple measurements of the local (Earth’s) magnetic field are used to derive *T* and *h*. Note that the Earth’s magnetic field varies with location and time and its value (magnitude and direction) is only approximately known by magnetic models such as International Geomagnetic Reference Field model (IGRF) [15]. However it is reasonable to assume that the magnitude of the magnetic field is (locally) constant during the calibration procedure. Based on this fact, most authors formulate an optimization or an estimation problem to derive *T* and *h*.

## 3. Alonso and Shuster (TWOSTEP)

The TWOSTEP algorithm [1] consists of an analytical centering approach [16,17] for its first step, while in the second step the solution is optimized numerically. The authors initially solved the problem of bias, *h*, determination when attitude is not known [18] and then extended their method to determine matrix *T* as well [1].

TWOSTEP is motivated by the assumption that matrix *T* should not be far from a pure rotation. Therefore by applying polar decomposition it can be written as $T={\left(I_{3\times 3}+D\right)}^{-1}O$ where *O* is an orthogonal matrix and *D* is a symmetric 3×3 matrix so as ${\left(I_{3\times 3}+D\right)}^{-1}$ to be positive definite. Matrix *O* can be integrated into vector *m* since it does not alter its norm. The equivalent measurement model is

(3) $$y=\hat{T}\hat{m}+h+\varepsilon$$

where

$$\begin{array}{cc} \hat{T} & ≜{\left(I_{3\times 3}+D\right)}^{-1}\\ \hat{m} & ≜Om. \end{array}$$

Therefore, for the full calibration, *D* and *h* must be estimated. To this purpose, a set of *N* measurements, $y_{k}$, k=1,2,⋯N, is used and the corresponding effective measurements $z_{k}$, k=1,2,⋯N, are defined as

(4) $$\begin{array}{cc} z_{k} & ≜{∥y_{k}∥}^{2}-{∥\hat{m_{k}}∥}^{2}\\ \; & ={∥y_{k}∥}^{2}-{∥m_{k}∥}^{2}. \end{array}$$

The last ones can be decomposed into a deterministic part plus an approximately Gaussian noise term, $\upsilon_{k}$ with mean $\mu_{k}$ and variance $\sigma_{k}^{2}$, i.e., $\upsilon_{k}∼N\left(\mu_{k},\;\sigma_{k}^{2}\right)$, given by

(5a) $$\begin{array}{cc} \mu_{k} & =-3\sigma^{2} \end{array}$$

(5b) $$\begin{array}{cc} \sigma_{k}^{2} & =4\sigma^{2}{\left(\left(I_{3\times 3}+D\right)y_{k}-h\right)}^{T}\left(\left(I_{3\times 3}+D\right)y_{k}-h\right)+6\sigma^{4}. \end{array}$$

Since *D* and *h* are unknown, the variance $\sigma_{k}^{2}$ is assumed to be similar to measurement’s output error variance $\sigma^{2}$. Hence $\mu_{k}$ and $\sigma_{k}^{2}$ can be assumed independent of *k*. To estimate *D* and *h*, Alonso and Shuster define the auxiliary quantities $E≜D^{2}+2D$ and c≜(I+D)h, as well as the estimation vector $\theta^{\prime }$ containing the elements of the 3×1 vector *c* and those of the 3×3 symmetric matrix *E*, which is formed as $\theta^{\prime }={\left[c^{T}E_{11}\;E_{22}\;E_{33}\;E_{12}\;E_{13}\;E_{23}\right]}^{T}$.

TWOSTEP algorithm functions on the estimation vector $\theta^{\prime }$ and thus on the auxiliary parameters, *E* and *c* and not on the actual calibration parameters, *D* and *h*. The transformation from *E* and *c* back to *D* and *h* is described in (13) and (14).

### 3.1. Initial Estimate

For every measurement, $y_{k}$, k=1,2,⋯N, the following auxiliary variables are defined

(6a) $$\begin{array}{cc} S_{k} & =[y_{k,1}^{2}\;y_{k,2}^{2}\;y_{k,3}^{2}\;2y_{k,1}y_{k,2}\;2y_{k,1}y_{k,3}\;2y_{k,2}y_{k,3}] \end{array}$$

(6b) $$\begin{array}{cc} L_{k} & =[2y_{k}^{T}\;-\;S_{k}]. \end{array}$$

The centering approximation is done using the following weighted averages along with their corresponding centered values

(7a) $$\begin{array}{cccc} \bar{z} & ≜{\bar{\sigma }}^{2}\sum_{k=1}^{N}\frac{1}{\sigma_{k}^{2}}z_{k} & {\tilde{z}}_{k} & ≜z_{k}-\bar{z} \end{array}$$

(7b) $$\begin{array}{cccc} \bar{L} & ≜{\bar{\sigma }}^{2}\sum_{k=1}^{N}\frac{1}{\sigma_{k}^{2}}L_{k} & {\tilde{L}}_{k} & ≜L_{k}-\bar{L} \end{array}$$

(7c) $$\begin{array}{cccc} \bar{\mu } & ≜{\bar{\sigma }}^{2}\sum_{k=1}^{N}\frac{1}{\sigma_{k}^{2}}\mu_{k} & {\tilde{\mu }}_{k} & ≜\mu_{k}-\bar{\mu } \end{array}$$

where

$${\bar{\sigma }}^{2}≜{\left(\sum_{k=1}^{N}\frac{1}{\sigma_{k}^{2}}\right)}^{-1}.$$

The first estimation of $\theta^{\prime }$ is the centered one given by

(8a) $$\begin{array}{cc} {\tilde{\theta }}^{\prime } & ={\tilde{P}}_{\theta^{\prime }\theta^{\prime }}\sum_{k=1}^{N}\frac{1}{\sigma_{k}^{2}}\left({\tilde{z}}_{k}-{\tilde{\mu }}_{k}\right){\tilde{L}}_{k}^{T} \end{array}$$

(8b) $$\begin{array}{cc} {\tilde{P}}_{\theta^{\prime }\theta^{\prime }}^{-1} & =\sum_{k=1}^{N}\frac{1}{\sigma_{k}^{2}}{\tilde{L}}_{k}^{T}{\tilde{L}}_{k} \end{array}$$

with ${\tilde{P}}_{\theta^{\prime }\theta^{\prime }}$ denoting the centered covariance matrix and ${\tilde{F}}_{\theta^{\prime }\theta^{\prime }}$ denoting the centered Fischer information matrix.

### 3.2. Solution Improvement Step

The second step improves the previous estimate of vector θ, derived in (8), via Gauss-Newton method using the centered estimate ${\tilde{\theta }}^{\prime }$ as the initial guess. The estimation is updated as follows

(9) $$\theta_{i+1}^{\prime }=\theta_{i}^{\prime }-{\left[{\tilde{F}}_{\theta^{\prime }\theta^{\prime }}+\frac{1}{{\bar{\sigma }}^{2}}{\left(\bar{L}-\phi \left(\theta_{i}^{\prime }\right)\right)}^{T}\left(\bar{L}-\phi \left(\theta_{i}^{\prime }\right)\right)\right]}^{-1}g\left(\theta_{i}^{\prime }\right)$$

where

(10a) $$\begin{array}{cc} v & ={\left(I_{3\times 3}+E\right)}^{-1}c \end{array}$$

(10b) $$\begin{array}{cc} \phi (\theta^{\prime }) & =[2v^{T}\;-v_{1}^{2}\;-v_{2}^{2}\;-v_{3}^{2}\;-2v_{1}v_{2}\;-2v_{1}v_{3}\;-2v_{2}v_{3}] \end{array}$$

(10c) $$\begin{array}{cc} g(\theta^{\prime }) & ={\tilde{P}}_{\theta^{\prime }\theta^{\prime }}^{-1}\left(\theta^{\prime }-{\tilde{\theta }}^{\prime }\right)-\frac{1}{{\bar{\sigma }}^{2}}\left(\bar{z}-\bar{L}\theta^{\prime }+c^{T}v-\bar{\mu }\right)\left({\bar{L}}^{T}-\phi \left(\theta^{\prime }\right)\right) \end{array}$$

with $v_{j}$ denoting the $j^{th}$ element of vector *v*. At every iteration the 3×3 symmetric matrix *E* and the 3×1 vector *c* are updated according to the current estimation vector $\theta_{i}^{\prime }$ using

(11) $$c=\left[\begin{array}{c} \theta_{1}^{\prime }\\ \theta_{2}^{\prime }\\ \theta_{3}^{\prime } \end{array}\right]\;\;\mathrm{and}\;\;E=\left[\begin{array}{ccc} \theta_{4}^{\prime } & \theta_{7}^{\prime } & \theta_{8}^{\prime }\\ \theta_{7}^{\prime } & \theta_{5}^{\prime } & \theta_{9}^{\prime }\\ \theta_{8}^{\prime } & \theta_{9}^{\prime } & \theta_{6}^{\prime } \end{array}\right].$$

Alonso and Shuster define the following quantity in order to establish a stop condition for the Gauss–Newton method.

(12) $$\eta_{i}≜{\left(\theta_{i+1}^{\prime }-\theta_{i}^{\prime }\right)}^{T}\left[{\tilde{F}}_{\theta^{\prime }\theta^{\prime }}+\frac{1}{{\bar{\sigma }}^{2}}{\left(\bar{L}-\phi \left(\theta_{i}^{\prime }\right)\right)}^{T}\left(\bar{L}-\phi \left(\theta_{i}^{\prime }\right)\right)\right]\left(\theta_{i+1}^{\prime }-\theta_{i}^{\prime }\right).$$

The iterations continue until $\eta_{i}$ became smaller than a predetermined threshold.

After sufficiently many iterations, an optimal estimation of matrix $E^{*}$ and of vector $c^{*}$ is derived. The derived solution is then used to find $D^{*}$ and $h^{*}$. To this end we apply SVD [19] to the symmetric matrix $E^{*}$, i.e.,

(13) $$E^{*}=USU^{T}$$

where $S=\mathrm{diag}(s_{1},s_{2},s_{3})$, U∈O(3). Then find the diagonal matrix $W=\mathrm{diag}(w_{1},w_{2},w_{3})$ satisfying $S=2W+W^{2}$. Typically, the elements of *S* are much smaller than unity [1] so a real solution exists with the diagonal entries of *W* being $w_{j}=-1+\sqrt{1+s_{j}}$, j=1,2,3.

Combining the above, the estimates of matrix $D^{*}$ and bias vector $h^{*}$ are given by

(14a) $$\begin{array}{cc} D^{*} & =UWU^{T} \end{array}$$

(14b) $$\begin{array}{cc} h^{*} & ={\left(I_{3\times 3}+D^{*}\right)}^{-1}c^{*} \end{array}$$

and are related to the calibration parameters *T* and *h* of the measurement model (2) as follows

(15) $$T={\left(I_{3\times 3}+D^{*}\right)}^{-1}\;\mathrm{and}\;h=h^{*}.$$

Summarizing, when the centered estimation is near the ground truth value the Gauss–Newton method typically converges rapidly. The authors verified the robustness of their method via simulations assuming either white noise or colored noise. TWOSTEP is also suitable for on-orbit calibration using IGRF data [15]. The algorithm is summarized in Algorithm 1.
**Algorithm 1:** Alonso and Shuster (TWOSTEP) [1]Step 1: Calculate $z_{k},L_{k},\;\mathrm{for}\;k=1,2,\cdots ,N$        by using (4)–(6)  Step 2: Calculate the centered values ${\tilde{z}}_{k},{\tilde{L}}_{k}\;\mathrm{for}\;k=1,2,\cdots ,N$ (7)Step 3: Calculate centered estimate ${\tilde{\theta }}^{\prime }$ and covariance matrix ${\tilde{P}}_{\theta^{\prime }\theta^{\prime }}$ (8)Step 4: Extract *c* and *E* from $\theta^{\prime }$ following (11)Step 5: Calculate $\phi (\theta^{\prime })$ and $g(\theta^{\prime })$ from (10)Step 6: Update $\theta^{\prime }$ using (9)Step 7: Calculate η following (12)Step 8: Repeat steps 4–7 until η is sufficiently small  Step 9: Apply SVD on $E^{*}$ (13) and define matrix *W*Step 10: Calculate $D^{*},h^{*}$ (14) and T,h (15)

## 4. Crassidis et al.

The authors of [6] were motivated by the fact that real-time applications demand real-time calibration methods. To this end, based on the problem formulation (3) established in TWOSTEP [1], Crassidis et al. formulate a real-time identification problem for the derivation of the calibration parameters *D* and *h* and solve it using the extended Kalman Filter (EKF) approach. Note that the authors of [6] have proposed two more algorithms dealing with the online calibration of a magnetic field sensor in [20,21]. However, in this work we focus on the EKF based one presented in [6] which is the most efficient and popular one.

Following the problem formulation of TWOSTEP, the bias vector *h* and the symmetric matrix *D* are desired. The estimation vector θ is defined differently and contains *h* and *D*, structured as follows

(16) $$\theta ={\left[h^{T}\;D_{11}\;D_{22}\;D_{33}\;D_{12}\;D_{13}\;D_{23}\right]}^{T}.$$

Because the vector θ is constant, the state model is given by $\dot{\theta }=0$. The effective measurement is given by $z_{k}={∥y_{k}∥}^{2}-{∥m_{k}∥}^{2}$ (4) while the measurement’s model is given by $z_{k}=\phi \left(\theta_{k}\right)+\upsilon_{k}$ where

(17) $$\phi \left(\theta_{k}\right)=-y_{k}^{T}\left(2D_{k}+D_{k}^{2}\right)y_{k}+2y_{k}^{T}\left(I_{3\times 3}+D_{k}\right)h_{k}-{∥h_{k}∥}^{2}$$

and effective measurement’s noise $\upsilon_{k}∼N\left(\mu_{k},\;\sigma_{k}^{2}\right)$ follows (5). At each iteration $D_{k}$ and $h_{k}$ are extracted from $\theta_{k}$ according to (16). The propagation is as it follows

(18a) $$\begin{array}{cc} \theta_{k+1} & =\theta_{k}+K_{k}\left[z_{k}-\phi \left(\theta_{k}\right)\right] \end{array}$$

(18b) $$\begin{array}{cc} P_{k+1} & =\left[I_{9\times 9}-K_{k}H\left(\theta_{k}\right)\right]P_{k} \end{array}$$

(18c) $$\begin{array}{cc} K_{k} & =P_{k}H^{T}\left(\theta_{k}\right){\left[H\left(\theta_{k}\right)P_{k}H^{T}\left(\theta_{k}\right)+\sigma_{k}^{2}\right]}^{-1} \end{array}$$

where $P_{k}$ is the covariance of the estimated parameters for *h* and *D* at step *k*. The matrix $H(\theta_{k})$ is the linearization matrix of $\phi (\theta_{k})$ and is defined as  

(19) $$H\left(\theta_{k}\right)=\left[2y_{k}^{T}\left(I_{3\times 3}+D_{k}\right)-2h_{k}^{T}\;\;\;\;-S_{k}F_{k}+2J_{k}\right]$$

where

(20a) $$S_{k}=\left[y_{k,1}^{2}\;\;y_{k,2}^{2}\;\;y_{k,3}^{2}\;\;2y_{k,1}y_{k,2}\;\;2y_{k,1}y_{k,3}\;\;2y_{k,2}y_{k,3}\right]$$

(20b) $$\begin{array}{cc} J_{k} & =[y_{k,1}h_{k,1}\;\;y_{k,2}h_{k,2}\;\;y_{k,3}h_{k,3}\;\;y_{k,1}h_{k,2}+y_{k,2}h_{k,1}\\ \; & \;\;\;y_{k,1}h_{k,3}+y_{k,3}h_{k,1}\;\;y_{k,2}h_{k,3}+y_{k,3}h_{k,2}] \end{array}$$

(20c) $$F_{k}=\left[\begin{array}{cccccc} \Delta_{1} & 0 & 0 & 2D_{k,12} & 2D_{k,13} & 0\\ 0 & \Delta_{2} & 0 & 2D_{k,12} & 0 & 2D_{k,23}\\ 0 & 0 & \Delta_{3} & 0 & 2D_{k,13} & 2D_{k,23}\\ D_{k,12} & D_{k,12} & 0 & \Delta_{4} & D_{k,23} & D_{k,13}\\ D_{k,13} & 0 & D_{k,13} & D_{k,23} & \Delta_{5} & D_{k,12}\\ 0 & D_{k,23} & D_{k,23} & D_{k,13} & D_{k,12} & \Delta_{6} \end{array}\right]$$

and

(21) $$\begin{array}{cc} \Delta_{1} & =2(1+D_{k,11})\\ \Delta_{2} & =2(1+D_{k,22})\\ \Delta_{3} & =2(1+D_{k,33})\\ \Delta_{4} & =2+D_{k,11}+D_{k,22}\\ \Delta_{5} & =2+D_{k,11}+D_{k,33}\\ \Delta_{6} & =2+D_{k,22}+D_{k,33}. \end{array}$$

  The noise variance of the measurements, $\sigma_{k}^{2}$, can be assumed to be constant and equal to $\sigma^{2}$ as in TWOSTEP. Given a set of *N* measurements, the EKF provides an optimal estimation vector $\theta^{*}=\theta_{N}$ from which an optimal vector $h^{*}=h_{N}$ and a matrix $D^{*}=D_{N}$ can be extracted according to (16). Therefore, the calibration parameters (2) are given by

(22) $$T={\left(I_{3\times 3}+D^{*}\right)}^{-1}\;\mathrm{and}\;h=h^{*}.$$

Under certain conditions, e.g., fast changing data, this approach of sequential calibration may have some advantages in terms of computational complexity and adaptation. The algorithm is summarized in Algorithm 2.
**Algorithm 2:** Crassidis et al. [6]Step 1: Initialize θ and k=0Step 2: **for each** measurement do:                Calculate $z_{k}$ (4)                Extract $D_{k}$ and $h_{k}$ from $\theta_{k}$ (16)                Calculate $S_{k},J_{k},F_{k}$ (20) and $H(\theta_{k})$ (19)                Calculate Kalman Gain $K_{k}$ (18)                Update estimation: $\theta_{k}\leftarrow \theta_{k+1}$                Update covariance matrix: $P_{k}\leftarrow P_{k+1}$ (18)                k←k+1Step 3: Extract $D^{*}$ and $h^{*}$ from $\theta^{*}$ (16)Step 4: Calculate *T* and *h* (22)

## 5. Dorveaux et al.

An iterative algorithm for the calibration of magnetic field sensors based on iterations of a least-squares problem is introduced in [3]. In the beginning of the algorithm, the measurements lie on an ellipsoid according to (2). In each iteration, the measurements move from the initial ellipsoid to the unit sphere, following a cost function minimization algorithm.

The authors in [3] use the following variation of the measurement model of (2)

(23) m=Ay+B

where $A=T^{-1}$, $B=-T^{-1}h$ and the measurement noise, ε, is neglected.

The algorithm begins by considering an initial estimate of the magnetic field vectors, denoted by ${\tilde{m}}_{k}\left(0\right)$ and defined as

(24) $${\tilde{m}}_{k}\left(0\right)=y_{k},\;k=1,2,\cdots ,K.$$

In every iteration, the following cost function is formulated and minimized using the least squares method.

(25) $$J\left(A,B,n\right)=\sum_{k=1}^{K}{∥A{\tilde{m}}_{k}\left(n\right)+B-\frac{{\tilde{m}}_{k}\left(n\right)}{∥{\tilde{m}}_{k}\left(n\right)∥}∥}^{2}$$

where n=1,2,⋯,N denotes the $n^{th}$ iteration. Let $A_{n}$ and $B_{n}$ be the resulting matrices from the minimization of (25). Every iteration ends with using $A_{n}$ and $B_{n}$ to update the estimates of the magnetic field vectors as

(26) $${\tilde{m}}_{k}\left(n+1\right)=A_{n}{\tilde{m}}_{k}\left(n\right)+B_{n},\;k=1,2,\cdots ,K.$$

From (26) we can express the magnetic field estimates ${\tilde{m}}_{k}\left(n\right)$ using the measurement vectors $y_{k}$ as

(27) $${\tilde{m}}_{k}\left(n\right)={\tilde{A}}_{n}y_{k}+{\tilde{B}}_{n},\;k=1,2,\cdots ,K$$

where ${\tilde{A}}_{n}$ and ${\tilde{B}}_{n}$ are iteratively defined as

(28) $${\tilde{A}}_{n}=A_{n}{\tilde{A}}_{n-1}\;\mathrm{and}\;{\tilde{B}}_{n}=A_{n}{\tilde{B}}_{n-1}+B_{n}.$$

To determine when the algorithm has reached an acceptable solution, we define the following cost

(29) $$J_{stop}\left(A_{n},B_{n}\right)=∥B_{n}∥+∥A_{n}-I_{3\times 3}∥.$$

The iterations stop when $J_{stop}$ is sufficiently small. Note that the original manuscript does not provide an explicit condition to stop iterations. However it is reasonable to terminate the algorithm when contribution of the updated $A_{n}$ and $B_{n}$ to the calibration parameters ${\tilde{A}}_{n}$ and ${\tilde{B}}_{n}$ is negligible (see (28)). The estimate $\tilde{m}\left(N\right)$ derived at the *N*th iteration represents the calibrated data and it is:

(30) $$m_{k}={\tilde{m}}_{k}\left(N\right),\;k=1,2,\cdots ,K$$

The derived matrices ${\tilde{A}}_{N}$ and ${\tilde{B}}_{N}$ are related to the calibration parameters *T* and *h* of the measurement model (2) as follows

(31) $$T={\tilde{A}}_{N}^{-1}\;\mathrm{and}\;h=-{\tilde{A}}_{N}^{-1}{\tilde{B}}_{N}.$$

Finally, the estimates ${\tilde{m}}_{k}\left(N\right)$, k=1,2,⋯,K, derived at the $N^{th}$ iteration represent the calibrated measurement vectors. The algorithm is summarized in Algorithm 3.
**Algorithm 3:** Dorveaux et al. [3]Step 1: Initialize ${\tilde{m}}_{k}\left(0\right)$ using (24).Step 2: Minimize (25) using least squares and derive $A_{n}$ and $B_{n}$.Step 3: Use $A_{n}$ and $B_{n}$ to calculate ${\tilde{m}}_{k}\left(n+1\right)$ from (26).Step 4: Calculate ${\tilde{A}}_{n}$ and ${\tilde{B}}_{n}$ using (28).Step 5: Evaluate the cost function $J_{stop}\left(A_{n},B_{n}\right)$ from (29).Step 6: Repeat steps 2-5 until $J_{stop}$ is sufficiently small.Step 7: Use ${\tilde{A}}_{N}$ and ${\tilde{B}}_{N}$ to calculate *T* and *h* using (31).

## 6. Vasconcelos et al.

The authors of [2] consider that magnetometers’ measurements lie on a ellipsoid manifold following the measurement model (2). First, they derive an initial estimate of the calibration parameters *T* and *h* by finding the ellipsoid that fits best to the given data. Then, they use the measurement model of (2) to formulate a maximum likelihood estimation problem and derive an improved estimate of the calibration parameters *T* and *h*.

From (2), the magnetic field vector is expressed as $m=T^{-1}\left(y-h\right)-T^{-1}\varepsilon$. Assuming that the magnitude of the magnetic field is constant during the calibration procedure we can write the following unconstrained optimization problem to derive *T* and *h*

(32) $$\begin{array}{cccc} \; & \underset{T,h}{\mathrm{minimize}}\; & \; & \sum_{k=1}^{K}{\left(\frac{∥T^{-1}\left(y_{k}-h\right)∥-1}{\sigma_{k}}\right)}^{2}. \end{array}$$

Here $\sigma_{k}$ denotes the standard deviation of the measurement noise in the $k^{th}$ measurement, assuming it is the same for all three axes and equal to σ. Without loss of generality, the magnitude of the magnetic field is assumed to be equal to one. A possible relaxation of this soft assumption is provided by Springmann [22] who addresses the problem of time-varying bias. To solve (32), the authors define the following cost function and then minimize it using the Newton’s method

(33) $$J\left(x\right)≜\sum_{k=1}^{K}{\left(\frac{∥\hat{T}\left(y_{k}-h\right)∥-1}{\sigma_{k}}\right)}^{2}$$

where $\hat{T}=T^{-1}$ and

(34) $$x={\left[\begin{array}{cc} \mathrm{vec}{\left(\hat{T}\right)}^{T} & h^{T} \end{array}\right]}^{T}.$$

The vector *x* is updated in every Newton’s iteration as follows

(35) $$x^{\left(+\right)}=x^{\left(-\right)}-{\left[\nabla^{2}J\left(x\right){|}_{x=x^{\left(-\right)}}\right]}^{-1}\nabla J\left(x\right){|}_{x=x^{\left(-\right)}}$$

where ∇J(x) is the gradient vector and $\nabla^{2}J\left(x\right)$ is the Hessian matrix of the cost function. For both ∇J(x) and $\nabla^{2}J\left(x\right)$, the authors in [2] provide analytical expressions which are presented in Appendix A.1.

### Initial Estimate

Solving (32) using the Newton’s method requires a good initial estimate of the calibration parameters, $\hat{T}$ and *h*. Vasconcelos et al. use a previous work on nonlinear estimators for strapdown magnetometers by Foster and Elkaim [23,24], to derive a good initial estimate. Solving the ellipsoid equation $∥m_{k}∥=∥T^{-1}\left(y_{k}-h\right)∥=1$ for every *k* is equivalent to solving the following pseudo-linear least squares estimation problem by re-arranging the terms as follows

(36) Lp=b

where, by writing each measurement vector as $y_{k}={\left[\begin{array}{ccc} y_{k}^{x} & y_{k}^{y} & y_{k}^{z} \end{array}\right]}^{T}$, k=1,2,⋯,K, it is

(37) $$L=\left[\begin{array}{ccccccccc} {y_{1}^{x}}^{2} & y_{1}^{x}y_{1}^{y} & y_{1}^{x}y_{1}^{z} & {y_{1}^{y}}^{2} & y_{1}^{y}y_{1}^{z} & y_{1}^{x} & y_{1}^{y} & y_{1}^{z} & 1\\ \vdots & \vdots & \vdots & \vdots & \vdots & \vdots & \vdots & \vdots & \vdots \\ {y_{K}^{x}}^{2} & y_{K}^{x}y_{K}^{y} & y_{K}^{x}y_{K}^{z} & {y_{K}^{y}}^{2} & y_{K}^{y}y_{K}^{z} & y_{K}^{x} & y_{K}^{y} & y_{K}^{z} & 1 \end{array}\right]$$

and

(38) $$b={\left[\begin{array}{cccc} {y_{1}^{z}}^{2} & {y_{2}^{z}}^{2} & \cdots & {y_{K}^{z}}^{2} \end{array}\right]}^{T}.$$

The vector *p* is derived as

(39) $$p={\left[\begin{array}{ccccccccc} A & B & C & D & E & G & H & I & J \end{array}\right]}^{T}={\left(L^{T}L\right)}^{-1}L^{T}b.$$

The initial estimates of the calibration parameters are derived as

(40) $$\hat{T}\left(0\right)=\left[\begin{array}{ccc} \frac{1}{\alpha } & 0 & 0\\ -\frac{1}{\alpha }\tan\left(\rho \right) & -\frac{1}{b}\sec\left(\rho \right) & 0\\ \frac{1}{\alpha }\left(\tan(\rho )\tan(\lambda )\sec(\phi )-\tan(\phi )\right) & -\frac{1}{b}\sec\left(\rho \right)\tan\left(\lambda \right)\sec\left(\phi \right) & \frac{1}{c}\sec\left(\lambda \right)\sec\left(\phi \right) \end{array}\right]$$

and

(41) $$h\left(0\right)=\frac{1}{2\alpha_{1}}{\left[\begin{array}{ccc} \beta_{1} & \beta_{2} & \beta_{3} \end{array}\right]}^{T}$$

where

(42) $$\begin{array}{cc} a & =\frac{1}{2\alpha_{1}}{\left(-\left(4D+E^{2}\right)\alpha_{2}\right)}^{1/2}\\ b & =\frac{1}{2\alpha_{1}}{\left(-\left(4A+C^{2}\right)\alpha_{2}\right)}^{1/2}\\ c & =\frac{1}{2\alpha_{1}}{\left(\left(4DA-B^{2}\right)\alpha_{2}\right)}^{1/2}\\ \tan(\rho ) & =-\frac{1}{2\alpha_{1}}\left(2B+EC\right){\left(\alpha_{1}\right)}^{-1/2}\\ \tan(\phi ) & =\left(BE-2CD\right){\left(\alpha_{1}\right)}^{-1/2}\\ \tan(\lambda ) & =E{\left(-\alpha_{1}\alpha_{3}^{-1}\right)}^{1/2} \end{array}$$

and

(43) $$\begin{array}{cc} \beta_{1} & =2BH+BEI-2CDI-4DG+ECH-E^{2}G\\ \beta_{2} & =-2AEI+4AH-BCI-2BG+C^{2}H-CEG\\ \beta_{3} & =4DIA-2DGC+EGB-IB^{2}-2EHA+CBH. \end{array}$$

The auxiliary variables $\alpha_{1}$, $\alpha_{2}$ and $\alpha_{3}$ are defined as

(44) $$\begin{array}{cc} \alpha_{1} & =-B^{2}+DC^{2}+4DA+AE^{2}-BEC\\ \alpha_{2} & =4AE^{2}J-E^{2}G^{2}-4BECJ+2ECHG+2BEIG-4EHAI-4DICG-C^{2}H^{2}\\ & +4DAI^{2}+2CBHI-4DG^{2}+4DC^{2}J+4BHG-4AH^{2}-B^{2}I^{2}-4B^{2}J+16DAJ\\ \alpha_{3} & =E^{4}A-CBE^{3}+E^{2}C^{2}D-2B^{2}E^{2}+8DAE^{2}-4DB^{2}+16D^{2}A. \end{array}$$

One contribution of Vasconcelos et al., advancing the existing initial step approach suggested in [23], was the derivation of the aforementioned explicit and non-trivial expressions. In addition, Vasconcelos et al. state that their proposed algorithm is applicable even when the magnitude of the magnetic field is not constant during the measurement, similarly to TWOSTEP and Crassidis et al. algorithm [6]. The algorithm is summarized in Algorithm 4.
**Algorithm 4:** Vasconcelos et al. [2]**Initial Estimate**Step 1: Use the sensors’ measurements $y_{k}$, k=1,2,⋯,K and form *A* and *b* according to (37) and (38), respectively.Step 2: Calculate *p* using (39)Step 3: Derive the initial estimates $\hat{T}\left(0\right)$ and h(0) using (40) and (41), respectively.**Newton Method**Step 4: Use the initial estimates $\hat{T}\left(0\right)$ and h(0) to initialize *x* according to (34).Step 5: Update *x* using (35).Step 6: Evaluate the cost function J(x) of (33).Step 7: Repeat Steps 5–6 until J(x) becomes sufficiently small.Step 8: Split *x* into $\hat{T}$ and *h* and calculate $T={\hat{T}}^{-1}$.

## 7. Ali et al.

The authors in [7] propose a Particle Swarm Optimization (PSO) [25] - based calibration algorithm that estimates the bias, the scale and nonorthogonality factors. The main advantage of this algorithm is its simplicity of implementation since the optimization is heuristic and does not depend on calculation of gradients, unlike other optimization techniques mentioned in this paper. It can be classified as an AI [26] approach.

The authors in [7] use (2) and a set of *N* sensor’s measurements to form the following optimization problem for deriving the calibration parameters *T* and *h*

(45) $$\underset{T,h}{\mathrm{minimize}}\sqrt{\sum_{k=0}^{N}{\left({∥y_{k}∥}^{2}-{∥m_{k}∥}^{2}\right)}^{2}}.$$

where $J≜\sqrt{\sum_{k=0}^{N}{\left({∥y_{k}∥}^{2}-{∥m_{k}∥}^{2}\right)}^{2}}$ is called the fitness value.

Function *J* depends on *T* and *h* which are conveniently combined into the single vector $x\in R^{12}$,

(46) $$x=\left[\begin{array}{c} h\\ \mathrm{vec}(T^{T}) \end{array}\right].$$

For a swarm of *S* particles, the position $x_{i}\in R^{12}$ and the velocity $v_{i}\in R^{12}$ of the $i^{th}$ particle can be computed using [25]

(47a) $$v_{i}^{k}=v_{i}^{k-1}+c_{1}r_{1i}^{k-1}\left(p_{i}^{k-1}-x_{i}^{k-1}\right)+c_{2}r_{2i}^{k-1}\left(p_{g}^{k-1}-x_{i}^{k-1}\right)$$

(47b) $$x_{i}^{k}=x_{i}^{k-1}+v_{i}^{k}$$

for i=1,2,⋯,S where *k* denotes the new value while k−1 the old value. Also $p_{i}$ denotes the $i^{th}$’s particle best position, $p_{g}$ denotes the swarm’s best position, $c_{1}$ and $c_{2}$ are the acceleration coefficients, *w* is the inertial weight factor and $r_{1i},r_{2i}$ are random numbers uniformly distributed within the range [0,1]. Typical values of these quantities are $c_{1}=c_{2}=2$, w=1 and the number of particles *S* is usually between 20 and 65.

Therefore, at each iteration *k*, each particle’s fitness value $J(x_{i}^{k})$ is calculated and quantities $p_{i}$ and $p_{g}$ are updated accordingly. The authors suggest three different stop criteria. Specifically, the iterations stop either when the fitness value *J* of a particle is smaller than a predetermined threshold, or after a maximum number of iterations, or when the change of *J* becomes insignificant with iterations. Upon termination of the algorithm, parameters *T* and *h* (2) are extracted from the swarms’s optimal solution $p_{g}$ according to

(48) $$\left[\begin{array}{c} h\\ \mathrm{vec}(T^{T}) \end{array}\right]=p_{g}.$$

Following the general concept of applying AI optimization algorithms, as was introduced in [7], one can also consider using more modern versions of the standard PSO, e.g., [27,28,29]. They are typically found as built-in functions in computational suites such as MATLAB [30]. The algorithm is summarized in Algorithm 5.
**Algorithm 5:** Ali et al. [7]Step 1: Initialize $x_{i},v_{i}$ for i=1,2,⋯,S        and set $p_{i}=x_{i}$Step 2: Find $j=\left\{i|i=1,2,\cdots ,S\;\mathrm{and}\;J(p_{i})\leftarrow \min\right\}$        Particle *i* best: $J_{min}^{i}\leftarrow J\left(p_{i}\right)$        Global best: $p_{g}\leftarrow p_{j}$ and $J_{min}\leftarrow J\left(p_{j}\right)$Step 3: **for each** particle *i* do                Update $x_{i},v_{i}$ (47)                Calculate $J(x_{i})$ (45)                **if** $J\left(x_{i}\right)<J_{min}^{i}$                   $J_{min}^{i}\leftarrow J\left(x_{i}\right)$ and $p_{i}\leftarrow x_{i}$                   **if** $J\left(x_{i}\right)<J_{min}$                       $J_{min}\leftarrow J\left(x_{i}\right)$ and $p_{g}\leftarrow x_{i}$Step 4: Repeat Step 3 until an exit condition is metStep 5: Extract *T* and *h* from $p_{g}$ (48)

## 8. Wu and Shi

The authors of [4], formulate the calibration of a three-axis magnetometer as a maximum likelihood estimation problem which is solved using the Gauss-Newton method.

Starting from the measurement model of (2), Wu and Shi observed that by considering the QR decomposition $T^{-1}=QR$, where Q∈O(3) and R∈U(3), (2) is written as

(49) $$y=R^{-1}Q^{T}m+h+\varepsilon .$$

Defining $\hat{m}≜Q^{T}m$, we observe that $∥\hat{m}∥=∥m∥$ since Q∈O(3). Also setting $\hat{T}≜R^{-1}$ we have that

(50) $$y=\hat{T}\hat{m}+h+\varepsilon .$$

Using the above transformation, the authors reduce the unknown model parameter variables from 12 (9 for *T* and 3 for *h*) to 9 (6 for *R* since *R* is upper triangular and 3 for *h*). Note that using (50), the calibration procedure now aims at finding the calibration parameters $\hat{T}$ and *h* while the magnetic field vector $\hat{m}$ is also unknown.

Using a set of *K* measurements and (50), the authors formulate the following maximum likelihood estimation problem

(51) $$\begin{array}{cccc} \; & \underset{\hat{T},h,{\hat{m}}_{k}}{\mathrm{minimize}}\; & \; & \sum_{k=1}^{K}∥y_{k}-\hat{T}{\hat{m}}_{k}{-h∥}^{2}\\ \; & \mathrm{subject}\;\mathrm{to}\; & \; & ∥{\hat{m}}_{k}∥=1,\;k=1,2,\ldots ,K. \end{array}$$

Without loss of generality, the authors, constrained the magnitude of the magnetic field to be equal to one. Based on (51), the following Lagrange function is formulated

(52) $$J\left(x\right)=\sum_{k=1}^{K}\left[∥y_{k}-\hat{T}{\hat{m}}_{k}{-h∥}^{2}+\lambda_{k}\left(∥{\hat{m}}_{k}{∥}^{2}-1\right)\right]$$

where

(53) $$x={\left[\mathrm{vec}{\left(\hat{T}\right)}^{T},\;h^{T},\;{\hat{m}}_{1}^{T},\;{\hat{m}}_{2}^{T},\;\ldots ,\;{\hat{m}}_{K}^{T},\;\lambda_{1},\;\lambda_{2},\;\ldots ,\;\lambda_{K}\right]}^{T}$$

and $\lambda_{k}$, k=1,2,⋯,K are positive Lagrange coefficients for the unit norm constrain. Note that since $\hat{T}$ is an upper triangular matrix, the lower triangular elements of $\hat{T}$ are excluded from *x*. The minimization of (52) and the estimation of *x* are done using the Gauss-Newton method as follows

(54) $$x^{\left(+\right)}=x^{\left(-\right)}-{\left[\nabla^{2}J\left(x\right){|}_{x=x^{\left(-\right)}}\right]}^{-1}\left(\nabla J\left(x\right){|}_{x=x^{\left(-\right)}}\right)$$

where ∇J(x) is the Jacobian vector and $\nabla^{2}J\left(x\right)$ is the Hessian matrix of the Lagrange function. For both ∇J(x) and $\nabla^{2}J\left(x\right)$, the authors provide analytical expressions which are presented in Appendix A.2.

### Initial Estimate

Solving (51) using the Gauss-Newton method requires a good initial estimate of the unknowns. To find one, the authors of [4] use the unit magnitude constrain and the equation $1=∥R\left(y_{k}-h\right){∥}^{2}$ which after some manipulation, is written as

(55) $$\left[\begin{array}{ccc} y_{k}^{T}⊗y_{k}^{T} & y_{k}^{T} & 1 \end{array}\right]\left[\begin{array}{c} \mathrm{vec}(A)\\ b\\ c \end{array}\right]≜Y_{k}z=0,\;k=1,2,\ldots ,K$$

where $A=R^{T}R$, $b=-2R^{T}Rh$ and $c=h^{T}R^{T}Rh$. Defining $Y={\left[\begin{array}{cccc} Y_{1}^{T} & Y_{2}^{T} & \cdots & Y_{K}^{T} \end{array}\right]}^{T}$, from (55) it is

(56) Yz=0

The authors, solve (56) using the least squares method and denote the solution $z_{e}={\left[\begin{array}{ccc} \mathrm{vec}{\left(A_{e}\right)}^{T} & b_{e}^{T} & c_{e} \end{array}\right]}^{T}=\min{∥Yz∥}^{2}$. They derive $z_{e}$ as the eigenvector of $Y^{T}Y$ corresponding to its minimum (or zero) eigenvalue. Using $z_{e}$, the vector *z* is derived as $z=\alpha z_{e}$, where $\alpha =4/\left(b_{e}^{T}A_{e}^{-1}b_{e}-4c_{e}\right)$. Extracting vec(A), *b* and *c* from *z*, the initial estimates of the unknowns, $\hat{T}\left(0\right),h\left(0\right),{\hat{m}}_{k}\left(0\right)$ and $\lambda_{k}\left(0\right)$ are defined as follows:

(57) $$\begin{array}{cc} \hat{T}\left(0\right) & =R^{-1}=\mathrm{chol}\left(A\right)\\ h(0) & =-A^{-1}b/2\\ {\hat{m}}_{k}\left(0\right) & =\hat{T}{\left(0\right)}^{-1}\left(y_{k}-h\right),\;k=1,2,\ldots ,K\\ \lambda_{k}\left(0\right) & =0,\;k=1,2,\ldots ,K \end{array}$$

where chol(·) is the Cholesky factorization.

Finally, an alternative version of Wu’s and Shi’s algorithm is proposed by Cao et al. in [13], where a different method for the initial estimate is presented, and the second step is identical. The algorithm is summarized in Algorithm 6.
**Algorithm 6:** Wu and Shi [4]**Initial Estimate**Step 1: Calculate $Y_{k}$, k=1,2,⋯,K from (55) and form the matrix $Y={\left[\begin{array}{cccc} Y_{1}^{T} & Y_{2}^{T} & \cdots & Y_{K}^{T} \end{array}\right]}^{T}$.Step 2: Find the eigenvector of $Y^{T}Y$ corresponding to its minimum (or zero) eigenvalue and denote it as $z_{e}={\left[\begin{array}{ccc} \mathrm{vec}{\left(A_{e}\right)}^{T} & b_{e}^{T} & c_{e} \end{array}\right]}^{T}$.Step 3: Calculate $z=az_{e}$ where $\alpha =4/\left(b_{e}^{T}A_{e}^{-1}b_{e}-4c_{e}\right)$.Step 4: Extract vec(A), *b* and *c* from *z*.Step 5: Calculate an initial estimate of the unknowns using (57).  **Gauss–Newton Method**Step 6: Use the initial estimates to initialize the vector *x* of (53)Step 7: Update *x* using (54).Step 8: Evaluate the cost J(x) of (52).Step 9: Repeat steps 7-8 until J(x) becomes sufficiently small.

## 9. Papafotis and Sotiriadis (MAG.I.C.AL.)

The authors in [5] use (2) and a set of *K* sensor’s measurements to form the following optimization problem for deriving the calibration parameters *T* and *h*

(58) $$\begin{array}{cccc} \; & \underset{T,h,m_{k}}{\mathrm{minimize}}\; & \; & \sum_{k=1}^{K}∥y_{k}-Tm_{k}{-h∥}^{2}\\ \; & \mathrm{subject}\;\mathrm{to}\; & \; & ∥m_{k}∥=1,\;k=1,2,\ldots ,K \end{array}$$

where, without loss of generality, the magnitude of the magnetic field is constrained to be equal to one. In order to solve (58) they propose an iterative algorithm, based on the solution of a linear least-squares problem.

The algorithm begins by initializing the magnetic field vectors, $m_{k}$, as 

(59) $$m_{k}=\frac{y_{k}}{∥y_{k}∥},\;k=1,2,\cdots ,K$$

and rewriting (2) in a matrix form as follows:

(60) Y=LG+E

where

(61a) $$\begin{array}{cc} Y & =\left[\begin{array}{cccc} y_{1} & y_{2} & \ldots & y_{K} \end{array}\right] \end{array}$$

(61b) $$\begin{array}{cc} L & =\left[\begin{array}{cc} T & h \end{array}\right] \end{array}$$

(61c) $$\begin{array}{cc} G & =\left[\begin{array}{cccc} m_{1} & m_{2} & \ldots & m_{K}\\ 1 & 1 & \ldots & 1 \end{array}\right] \end{array}$$

(61d) $$\begin{array}{cc} E & =\left[\begin{array}{cccc} \varepsilon_{1} & \varepsilon_{2} & \ldots & \varepsilon_{K} \end{array}\right]. \end{array}$$

In every iteration, (60) is solved for *L* using the least squares method, minimizing the total squared error $∥E^{T}{E∥}_{F}^{2}$

(62) $$L=YG^{T}{\left(GG^{T}\right)}^{-1}$$

From the calculated *L*, an updated set of calibration parameters *T* and *h* is extracted from (61b). Using them, the magnetic field vector is updated as

(63) $$m_{k}=\frac{\tilde{m_{k}}}{∥\tilde{m_{k}}∥},\;k=1,2,\cdots ,K$$

where

(64) $${\tilde{m}}_{k}=T^{-1}\left(y_{k}-h\right),\;k=1,2,\cdots ,K.$$

Every iteration ends by updating the matrix *G* using the updated vectors $m_{k}$, k=1,2,⋯,K. Iterations stop when a small value of the following cost function is achieved

(65) $$J\left(T,h\right)=\sum_{k=1}^{K}{\left(∥{\tilde{m}}_{k}{∥}^{2}-1\right)}^{2}.$$

MAG.I.C.AL. algorithm is summarized in Algorithm 7.   
**Algorithm 7:** Papafotis and Sotiriadis (MAG.I.C.AL.) [5]Step 1: Initialize $m_{k}$ using (59).Step 2: Calculate *L* using (62).Step 3: Extract *T* and *h* from *L* using (61b).Step 4: Update $m_{k}$ using (63) and (64) and use it to update *G*.Step 5: Evaluate the cost-plus-penalty function *J* from (65).Step 6: Repeat steps 2-5 until J(T,h) is sufficiently small.

## 10. Algorithm Evaluation and Comparison

In this Section, the performance of the presented algorithms are evaluated in terms of accuracy, robustness, and execution speed. Firstly, we evaluate the performance of the seven algorithms using multiple sets of synthetic data where the calibration parameters *T* and *h*, as well as the measurement noise characteristics are predefined and known. By doing so, we are able to accurately determine the algorithms’ accuracy and robustness. Then multiple datasets of two different low-cost magnetic field sensors are used to verify the algorithms’ performance under real-world conditions.

### 10.1. Synthetic Data Generation

We designed a procedure to generate synthetic data effectively, in order to examine each of the aforementioned algorithm’s performance across a range of noise variance and measurement sample size. The authors of TWOSTEP [18] propose a typical scenario of assuming the magnetic vector spinning with a constant angular velocity. On the other hand, Wu and Shi [4] suggest a specific sequence of 3D rotations using Euler Angles, applied on a constant known magnetic vector *m*. In the same page, Papafotis and Sotiriadis [5] recommend a sequence of 12 approximate orientations. Another alternative is to make use of a set of random, yet normalized, vector fields, which however demands a reasonable amount of samples.

Because none of the described algorithms guarantees that it will function properly under an arbitrary dataset, we propose an efficient method to span SO(3), following [31], so as to provide the algorithms with substantial, non-redundant information and to compare them fairly. After extensive simulation, it was observed that the recommended method was very effective in spanning the 3D rotation space.

Our method’s effectiveness lies in distributing the points on the sphere ∥m∥=1, more evenly by using the canonical Fibonacci lattice mapping [31,32]. Generating a Fibonacci sphere is an extremely fast and effective approximate method to evenly distribute points on a sphere.

This way SO(3) is sufficiently represented even with only a small dataset. An algorithm for generating *K* vectors distributed on a Fibonacci sphere is presented in detail in Algorithm 8.

Considering *K* vectors, $m_{k}$, k=1,2,⋯,K distributed on a Fibonacci sphere, we continue with generating matrix *T* and vector *h*, required to calculate the corresponding measurement vectors $y_{k}$, $m_{k}$, k=1,2,⋯,K according to (2). Ideally, matrix *T* would be the 3×3 identity matrix while the bias vector *h* would be the 3×1 vector of zeros. A realistic model for *T* and *h*, accounting for the sensor’s non-idealities, is derived by using the concept of additive perturbation

(66a) $$\begin{array}{cc} T & =\alpha I_{3\times 3}+E \end{array}$$

(66b) $$\begin{array}{cc} h & =e \end{array}$$

where α accounts for gross scaling errors, *E* is a 3×3 perturbation matrix with random, typically small, coefficients and *e* is 3×1 perturbation bias vector with random coefficients. Finally, a sequence of white noise $\varepsilon ∼N(0,\;\sigma^{2})$ is added to the measurements and the measurement vectors $y_{k}$, $m_{k}$, k=1,2,⋯,K are derived according to (2)

(67) y=Tm+h+ε.

**Algorithm 8:** Generation of synthetic data
Step 1:Initialize the number of measurements *K* and the radius of sphere *r*
Step 2: Calculate Golden Ratio: $\varphi =\frac{1+\sqrt{5}}{2}$
Step 3: **for each** k=1,2,⋯,K do:
        $\theta =\frac{2\pi k}{\varphi }$
        $\phi =\arccos\left(1-\frac{2(k-0.5)}{K}\right)$
        $m_{k}=\left[m_{x},m_{y},m_{z}\right]=\left[r\cos\theta \sin\phi ,r\sin\theta \sin\phi ,r\cos\phi \right]$
Step 4: Pick the scaling parameter, α, the perturbation matrix, *E*
        and the perturbation vector, *e*.
Step 5: Calculate *T* and *h* according to (66).
Step 6: Generate a sequence of white noise: $\varepsilon ∼N(0,\;\sigma^{2})$
Step 7: Calculate the measurement vectors: $y_{k}=Tm_{k}+h+\varepsilon_{k}$ (2)


**Figure 1:**
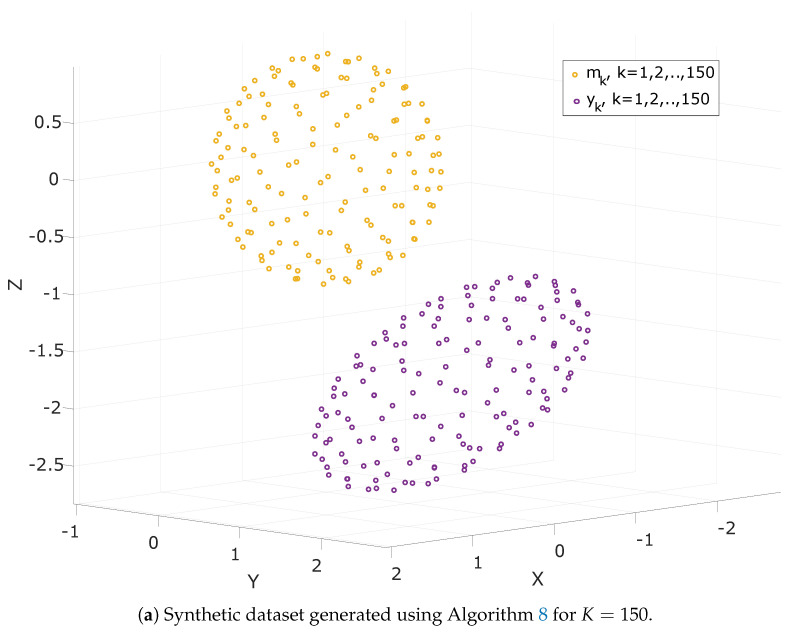

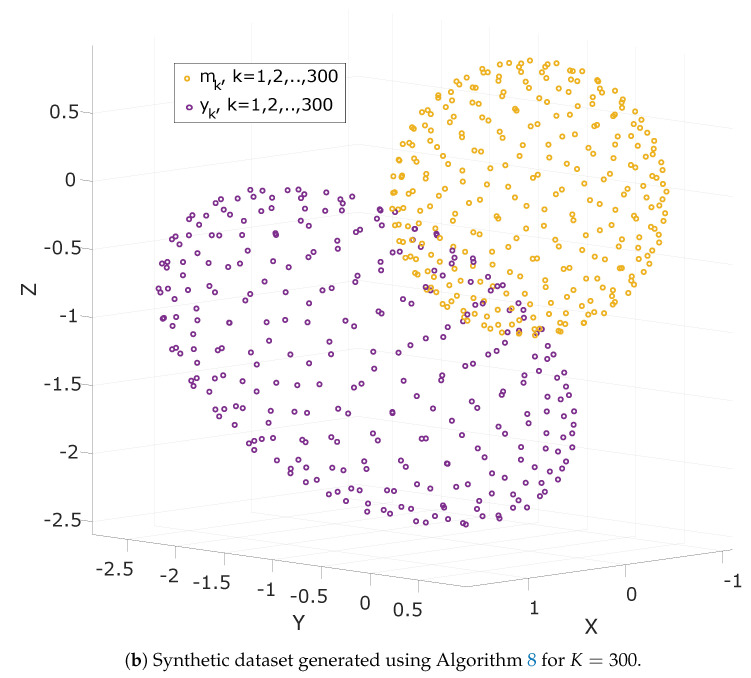
Two synthetic datasets generated using Algorithm 8 for K=150 (**a**) and K=300 (**b**), respectively.

The two datasets generated using Algorithm 8 are presented in Figure 1. Note that for visualization purposes, the scaling parameter, α, the perturbation matrix, *E*, and the perturbation vector, *e*, used to create each dataset were set to a rather large value.

#### 10.1.1. Experiment Setup and Evaluation Criteria

To evaluate the performance of the algorithms, we used synthetic data, generated by Algorithm 8, and we executed a great number Monte Carlo simulations. Each simulation consisted of 250 runs of each algorithm while in each run, the same dataset was used as input in all algorithms. An uncertainty was introduced in the generation of each dataset by considering a statistical distribution for the elements, $E_{ij}$, of the perturbation matrix, *E*, and the elements, $e_{i}$, of the perturbation vector *e* (see (66)). Specifically, for the Monte Carlo simulations we assumed

(68a) $$\begin{array}{cc} \alpha & ∼U[0.8,1.2] \end{array}$$

(68b) $$\begin{array}{cc} E_{ij} & ∼U[-\beta ,\beta ] \end{array}$$

(68c) $$\begin{array}{cc} e_{i} & ∼U[-\gamma ,\gamma ] \end{array}$$

where β and γ are scalars, the effect of which was tested using multiple Monte Carlo simulations. Note that we considered the scaling factor, α, to be close to the ideal value of α=1. That may not be the case when real-world measurements are used, however, it is trivial, and common, to properly scale the measurements before the calibration procedure and remove gross scaling errors. In this way, the algorithms are not burdened, searching for a scaling relationship which can be easily provided by simple data preprocessing.

A challenging point while setting up the experiments was to determine the number of samples of each dataset and the value of the sensor’s noise variance, $\sigma^{2}$. We considered a dataset of 300 measurements as a solid choice for a simulation environment based on [4,7] while we experimentally confirmed that bigger datasets do not improve the performance of any algorithm. We also examined the performance of the presented algorithms when smaller datasets, consisting of 150 and 50 measurements, are used. As far as the noise variance, $\sigma^{2}$, is concerned, we considered a nominal value of σ=0.005, following [2,4], while we also simulated the cases of more noisy (σ=0.05) and less noisy (σ=0.0005) sensors.

The evaluation of the algorithm for each Monte Carlo simulation was completed in terms of accuracy, execution speed, and robustness. We used the execution speed of each algorithm as a metric of computational efficiency and is defined as the inverse of the mean execution time. As a metric of robustness we considered the percentage of datasets for which each algorithm successfully derived a meaningful solution.

The definition of an accuracy metric is a little more involved. Each algorithm was developed to take as inputs the measurement vectors $y_{k}$, k=1,2,⋯,K and output the calibration parameters *T* and *h*. Comparing the output bias vector *h* with the true one, $h_{true}$, which was used in the data generation procedure, was performed by defining the following cost

(69) $$J_{h}=∥h_{true}-h∥.$$

The calibration matrix *T* on the other hand is derived under a rotational uncertainty and comparing it with the true one, $T_{true}$, is a more challenging task.

Consider the measurement model of (2). Noting that the true magnetic field vector in (2) is also unknown, and derived by the calibration algorithm, we can write:

(70) $$y=T_{true}RR^{T}m+h_{true}$$

where *R* is an orthogonal matrix in the O(3) group. Thus, taking into account the rotational invariance of the Euclidean norm which implies that $∥R^{T}m∥=∥m∥$, a calibration algorithm may output any matrix *T* of the form $T=T_{true}R$. Thus, a proper cost function to compare *T* and $T_{true}$ is the following

(71) $$J_{T}={∥T-T_{true}R∥}_{F}$$

where, the matrix *R* is defined as the solution of the following minimization problem

(72) $$R=\underset{\Omega \in O(3)}{\mathrm{argmin}}{∥T-T_{true}\Omega ∥}_{F}.$$

The solution of (72) is given by the orthogonal procrustes problem [33], and it is

(73) $$R=UV^{T}$$

where the matrices *U* and *V* are derived from the singular value decomposition (SVD) of the matrix $T_{true}^{T}T$, i.e., $T_{true}^{T}T=U\Sigma V^{T}$, where U,V∈O(3) and Σ is a diagonal matrix.

Using (69) and (71) we define the following cost function as a metric of accuracy

(74) $$J=∥h_{true}-h∥+∥T-T_{true}{R∥}_{F}.$$

Based on the above and given the results of a Monte Carlo simulation consisted of *N* executions of each algorithm, we define the following metrics of performance:

- **Accuracy** is defined as the mean value of the cost *J*, defined in (74), across all *N* executions with meaningful output.
- **Mean execution time** is defined as the mean value of the execution time of an algorithm.
- **Robustness** is defined as the percentage of datasets for which each algorithm successfully derived a meaningful solution.

The robustness criterion can be seen as the frequency in which an algorithm provides a better solution (T,h) in the sense of the cost function (74), than the trivial solution $\left(I_{3\times 3},0_{3\times 1}\right)$ which assumes no bias and non multiplicative errors. Given the cost $J_{o}$ that corresponds to the trivial solution,

(75) $$J_{o}=∥h_{true}-0_{3\times 1}∥+∥I_{3\times 3}-T_{true}{R∥}_{F}$$

an execution of an algorithm is considered as successful with meaningful output when

(76) $$J<\delta J_{o}$$

where δ∈(0,1) is a robustness parameter. If δ is close to 1, it means that only little improvement with respect to $J_{o}$ is sufficient. As δ gets smaller, better solutions are required. Thus, this parameter can be tuned with respect to the test’s objective and the application’s specifications. Given *N* runs for an algorithm, its robustness is denoted by RB(%) and is defined as

(77) $$RB\left(\%\right)=\frac{1}{N}\sum_{i=1}^{N}U(J_{i}<\delta J_{oi})\cdot 100.$$

Here $J_{i}$ and $J_{oi}$ are the values of *J* (74) and $J_{o}$ (75), respectively, corresponding to the *i*th run of the algorithm and U is a boolean function, which is one if its argument is true and zero otherwise. Let *M* denote the number of executions meaningful outputs.

Now, the accuracy metric is only applied on the *M* meaningful outputs according to the robustness test (76), since otherwise the comparison would be unfair for the least stable algorithms. The accuracy of an algorithm over a dataset is denoted by ρ and it is defined as

(78) $$\rho =\frac{1}{M}\sum_{i=1}^{N}U\left(J_{i}<\delta J_{oi}\right)J_{i}$$

which is the mean accuracy metric value over the *M* executions with meaningful outputs.

Similarly, the time-efficiency metric (i.e., mean execution time) is only applied on the *M* executions with meaningful outputs according to the robustness test (76). Again, this is because otherwise the comparison would be unfair for the least stable algorithms. The mean execution time of an algorithm over a dataset, is denoted by τ and is defined as

(79) $$\tau =\frac{1}{M}\sum_{i=1}^{N}U\left(J_{i}<\delta J_{oi}\right)t_{i}$$

where $t_{i}$ is the time needed for the *i* run to be completed. The execution speed of an algorithm is defined as 1/τ.

#### 10.1.2. Baseline Evaluation

To derive a baseline evaluation of the presented algorithms, we run a Monte Carlo simulation considering typical values for the sensor’s error and noise parameters. In this simulation we neglected the effect of hard-iron and soft-iron distortions which are, in some cases, the dominant terms of the overall error, as well as extreme cases of large manufacturing imperfections. More specifically, 250 different datasets consisting of 300 measurements each, were generated following Algorithm 8 and considering the following distributions of the model disturbances and the measurement noise

(80) $$\begin{array}{cc} \alpha & ∼U[0.8,1.2]\\ E_{ij} & ∼U[-0.05,0.05]\\ e_{i} & ∼U[-0.05,0.05]\\ \sigma & =0.005 \end{array}$$

The distribution ranges in (80) are based on our literature review. The selection β=γ=0.05 corresponds to the typical case of approximately 5% distortion for *T* and bias *h*. The measurement noise standard deviation is set to a typical value of σ=0.005 [2,4].

The performance of the seven algorithms is presented in Table 2.

#### 10.1.3. The Effect of the Offset Perturbation Parameter, γ

Under extreme manufacturing imperfections or the effect of hard-iron distortion, the magnitude of the offset vector, *h*, can be much larger than that in the typical case. In this Section, we examine how larger values of ∥h∥ affect the performance of the presented algorithms. To do so, we run six Monte Carlo simulations, each one comprised of 250 different datasets generated by following Algorithm 8. The offset vector perturbation parameter $e_{i}$ is simulated with gradually increasing magnitude by expanding the selection horizon U[−γ,γ]. Afterwards, its corresponding impact on each algorithm’s robustness and accuracy is investigated. The distributions of the model disturbances and measurement noise are:

$$\begin{array}{cc} \alpha & ∼U[0.8,1.2]\\ E_{ij} & ∼U[0.05,0.05]\\ e_{i} & ∼U[-\gamma_{l},\gamma_{l}]\\ \sigma & =0.005 \end{array}$$

for various γ

**Figure 2:**
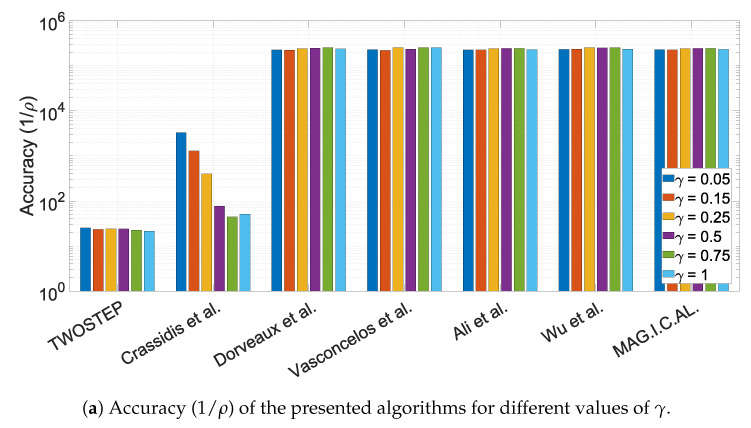

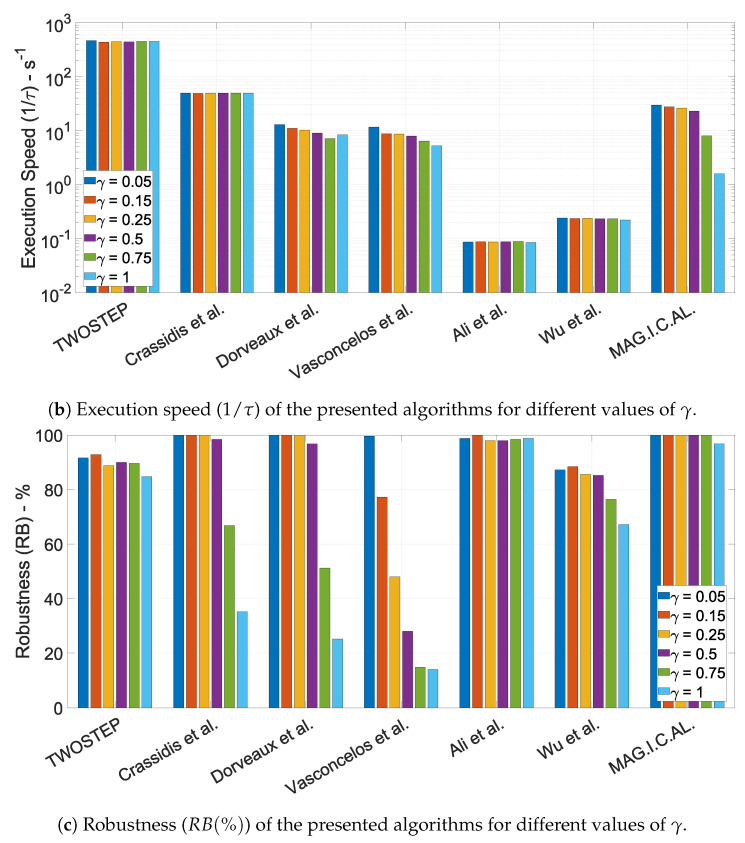
Performance characteristics of the presented algorithms for different values of γ.

γ={0.05,0.15,0.25,0.5,0.75,1}

where l=1,2,⋯,6 is the index of Monte Carlo simulation. The extreme case of γ=1 addresses the possibility of bias being clearly comparable and even indistinguishable to the true magnetic vector. Therefore, as γ increases, the algorithms were driven to their limits and their functionality range was identified. All the other parameters were nominal, to ensure a fair comparison. The results of the six Monte Carlo simulations are presented in Figure 2.

The MAG.I.C.AL and the PSO methods are the most robust ones since they function almost always, even for large values of bias, while TWOSTEP and Wu and Shi’s algorithms are a little less stable. In addition, Dorveaux et al. algorithm and EKF seem to be reliable for small to moderate values of bias. All algorithms, except TWOSTEP and EKF are extremely precise when they function properly. No changes in execution speed are noticed, with the exception of MAG.I.C.AL which probably requires more iterations as the bias increases.

#### 10.1.4. The Effect of the Calibration Matrix Perturbation Parameter, β

Similar to the case of the offset vector, *h*, under extreme manufacturing imperfections or the effect of soft-distortion, matrix *T*, can also diverge significantly from the typical case of the identity matrix. In this Section, we examine how larger values of perturbation *E* affect the performance of the presented algorithms. To do so, we run six Monte Carlo simulations, each one based on 250 different datasets generated by following Algorithm 8. The perturbation elements $E_{ij}$ were simulated with gradually increasing magnitude by expanding the distribution range U[−β,β]. Afterwards, its corresponding impact on each algorithm’s robustness and accuracy is investigated. The distributions of the model disturbances and measurement noise are:

$$\begin{array}{cc} \alpha & ∼U[0.8,1.2]\\ E_{ij} & ∼U[-\beta_{l},\beta_{l}]\\ e_{i} & ∼U[-0.05,0.05]\\ \sigma & =0.005 \end{array}$$

for various β

**Figure 3:**
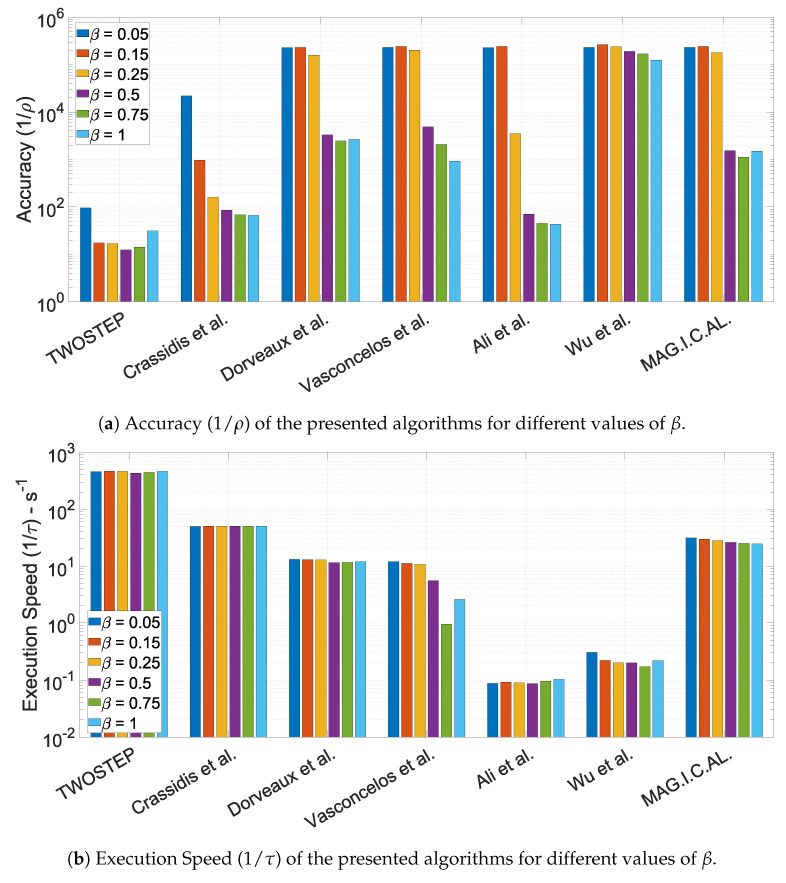

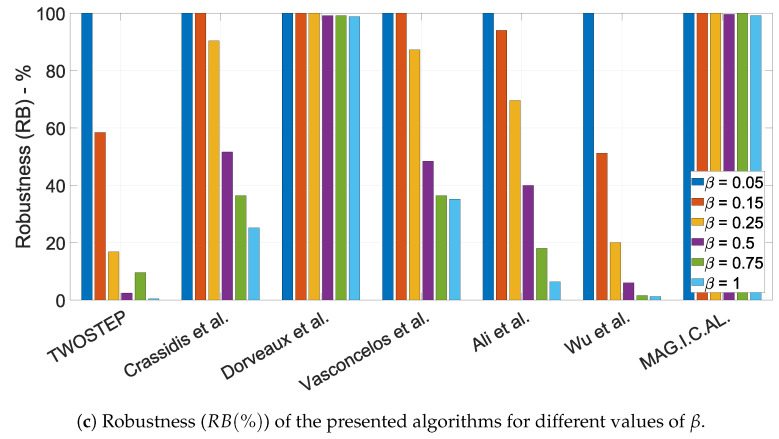
Performance characteristics of the presented algorithms for different values of β.

β={0.05,0.15,0.25,0.5,0.75,1}

where l=1,2,⋯,6 is the index of Monte Carlo simulation. As β increases, the algorithms were driven to their limits and their functionality range was identified. All the other parameters were nominal, to ensure a fair comparison. The results of the six Monte Carlo simulations are presented in Figure 3.

The MAG.I.C.AL algorithm and the algorithm of Dorveaux et al. appear to be the most robust and effective, with similar accuracy. The algorithm of Vasconcelos et al., the PSO algorithm and the EKF algorithm succeed only for small to moderate non-orthogonality errors. Vasconcelos et al. achieves accuracy comparable to that of MAG.I.C.AL. The rest of the algorithms tend to fail frequently as these errors increase. What is surprising is that Wu and Shi’s algorithm provides the most accurate solutions for all β values, but with very low robustness. To conclude, most algorithms handle bias distortion better than non-orthogonality errors.

#### 10.1.5. The Effect of Dataset Size, *K*

In this section, we examine how the dataset size, *K*, affects the algorithms’ performance. In general, the diversity of the measurement directions is more crucial than the quantity of them, e.g., a dataset of 50 measurements with directions distributed near uniformly on the unit sphere is significantly more suitable for the algorithms than one with thousands of measurements all having approximately the same direction.

According to existing literature [4,5,7], an order of 300 measurements with directions sufficiently covering the unit sphere form an acceptable dataset for the calibration. Here we use datasets with 50, 150, and 300 measurements to test the algorithms’ limits. To do so, we run three Monte Carlo simulations, based on 250 different datasets generated by Algorithm 8. The distributions of the model disturbances and measurement noise are:

$$\begin{array}{cc} \alpha & ∼U[0.8,1.2]\\ E_{ij} & ∼U[-0.05,0.05]\\ e_{i} & ∼U[-0.05,0.05]\\ \sigma & =0.005 \end{array}$$

**Figure 4:**
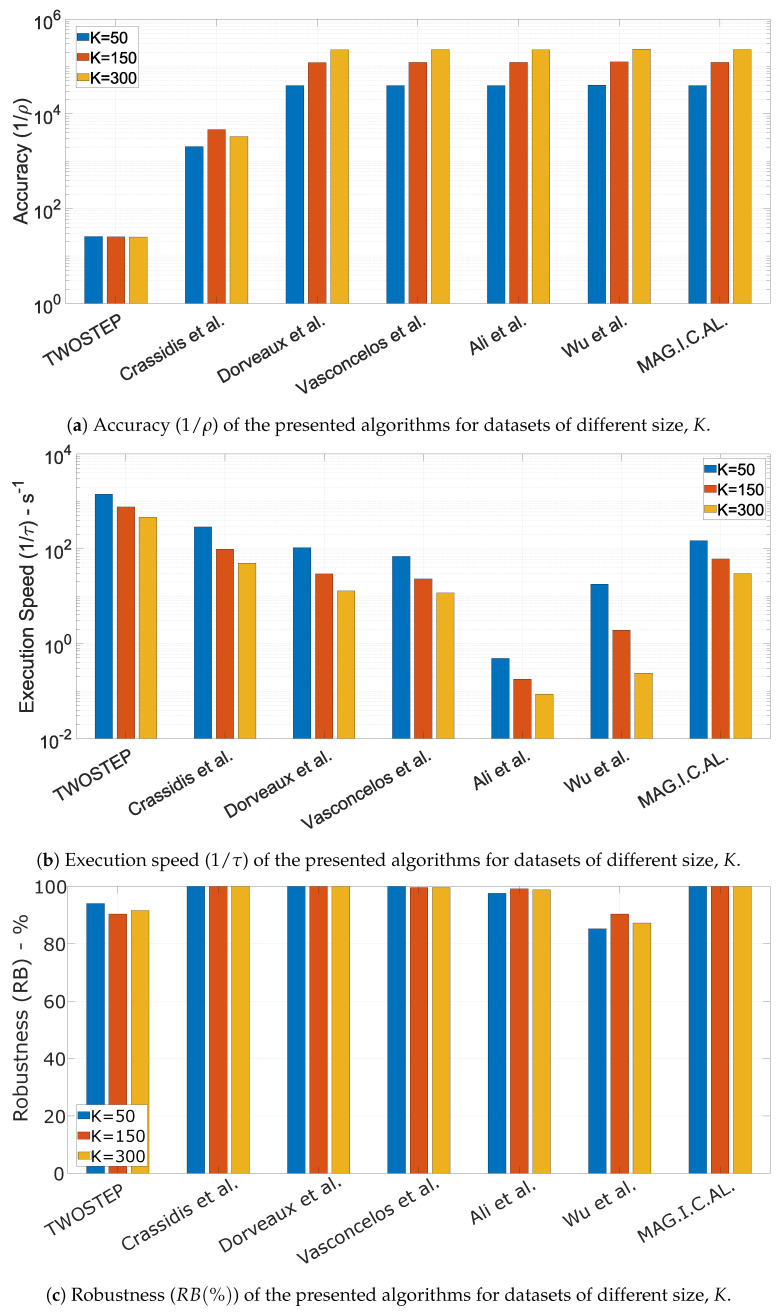
Performance characteristics of the presented algorithms for different values of *K*.

The dataset size *K* varied whereas the distributions’ ranges were fixed to nominal to ensure a fair comparison. The results of the three Monte Carlo simulations are presented in Figure 4.

In general, the dataset size, *K*, does not seem to be important in terms of robustness. Accuracy is surprisingly high even with only 50 measurements, which is probably an outcome of the well distributed measurement directions using the Fibonacci lattice. Furthermore, the algorithms execution time appeared to be linear with *K*.

#### 10.1.6. The Effect of the Noise Variance, $\sigma^{2}$

In this section, we examine the influence of measurement noise variance σ on algorithms’ robustness and accuracy. The assumption of pure white Gaussian noise in the measurement model was done. We considered a nominal value of σ=0.005, following [2,4], while we also simulated the cases of more noisy (σ=0.05) and less noisy (σ=0.0005) sensors. With these choices, we represented algorithms’ capabilities under 3 different orders in the magnitude of the error in the measurement. To do so, we run three Monte Carlo simulations, each one based on 250 different datasets generated by following Algorithm 8. The distributions of the model disturbances and measurement noise are:

$$\begin{array}{cc} \alpha & ∼U[0.8,1.2]\\ E_{ij} & ∼U[-0.05,0.05]\\ e_{i} & ∼U[-0.05,0.05]\\ \sigma & =\{0.0005,0.005,0.05\} \end{array}$$

**Figure 5:**
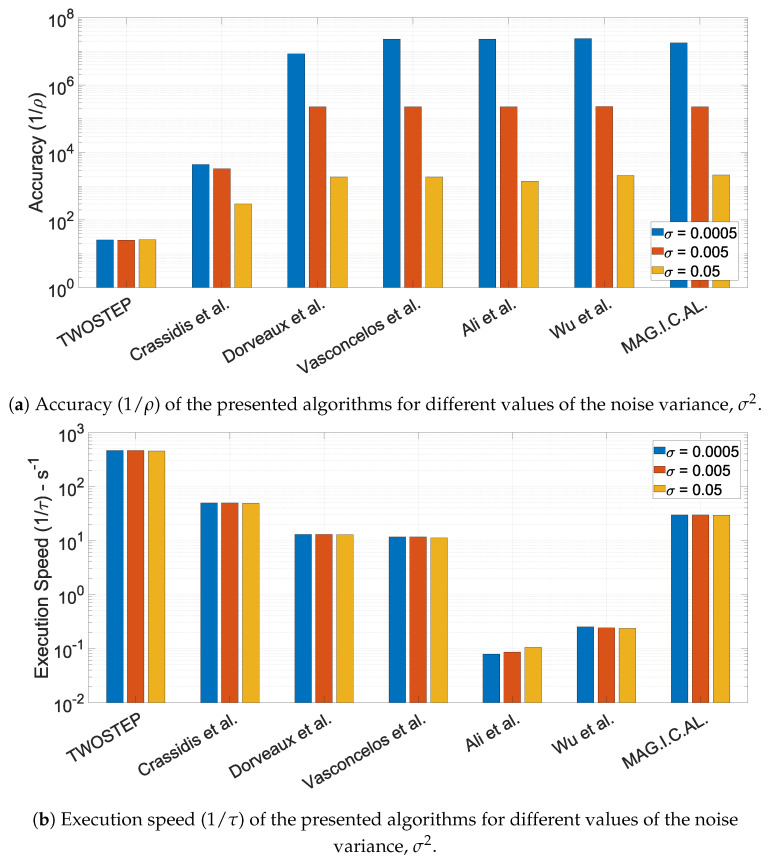

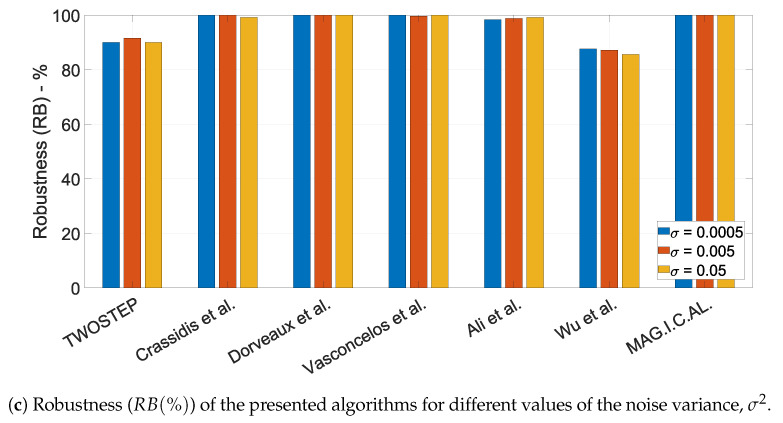
Performance characteristics of the presented algorithms for different values of the noise variance, $\sigma^{2}$.

Finally, all parameters except σ were set to their default ones, to ensure a fair comparison. The results of the three Monte Carlo simulations are presented in Figure 5.

All algorithms appear to be immune to the change of measurement’s output variance σ. What is worth mentioning is that an increase of one order in variance resulted to a decrease of one order in accuracy for most algorithms (i.e., MAG.I.C.AL, Ali et al., Vasconcelos et al., Dorveaux et al., Wu and Shi).

### 10.2. Algorithm Evaluation Using Real Data

In this section, the aforementioned algorithms are tested using real data. Multiple datasets captured by low-cost magnetic field sensors were used to verify the algorithms’ performance under real-world conditions. In this case, parameters $T_{true}$ and $h_{true}$ are not known in advance. Therefore, the accuracy metric (74) cannot be used. Since, the measurements took place in a specific location, a constant magnitude of magnetic vector, ∥m∥=1 was considered. As a result, a proper cost function to evaluate an algorithm’s effectiveness is the following

(81) $$J_{r}=\frac{1}{K}\sum_{i=1}^{K}{\left({∥m_{k}∥}^{2}-1\right)}^{2}$$

where *K* is the number of measurements and k=1,2,⋯,K is the measurement index. The estimated magnetic field vector $m_{k}$ for each *k* is given by

(82) $$m_{k}=T^{-1}\left(y_{k}-h\right)$$

where *T* and *h* are the outputs of a calibration algorithm. Such a cost function is described by Wu and Shi (52), as well as by Papafotis and Sotiriadis (65).

To evaluate the performance of the presented algorithms, we used two off-the-shelf, low-cost magnetic field sensors, which are typically found in commercial electronic devices, such as smartphones, activity trackers, etc. More specifically, we captured a total of 30 datasets using the LSM9DS1 by STMicroelectronics and the BNO055 by Bosch Sensortec. The operation parameters of the two sensors during the experiment are presented in Table 3.

**Figure 6:**
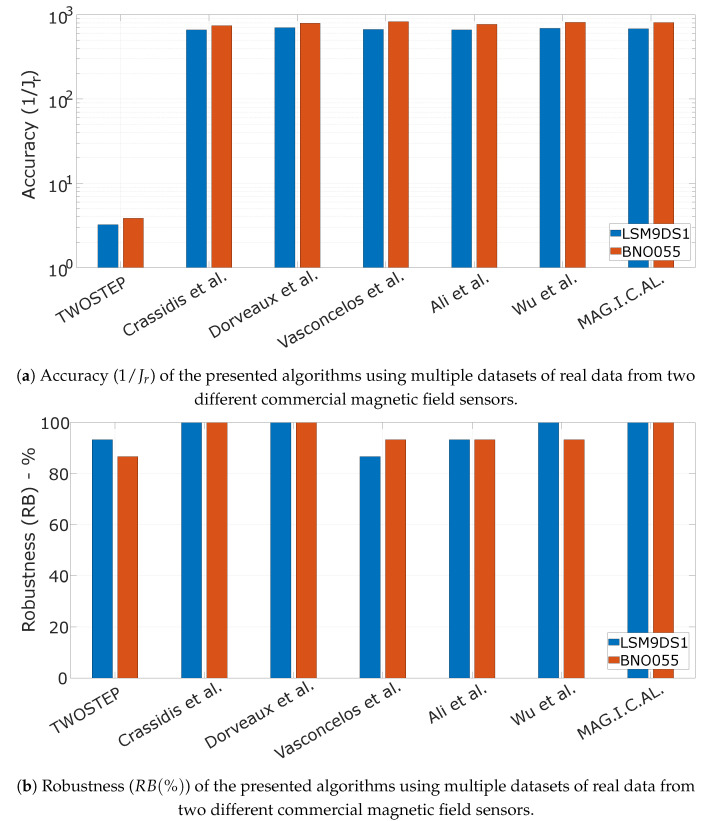
Performance characteristics of the presented algorithms using multiple datasets of real data from two different commercial magnetic field sensors.

During the experiment, two sensors were fixed on the same rigid platform which was rotated by hand in several orientations. In Figure 6a, the mean value of the cost function (81) across all the recorded datasets for every algorithm is presented as a metric of accuracy. The robustness of each algorithm, as defined in (77) is presented in Figure 6b. Note that both Figure 6a,b are in agreement with the results obtained in Section 10.1.2 where synthetic data with typical values for sensor’s noise and measurement distortion were considered.

## 11. Conclusions

To summarize, a complete and extensive study on calibration methods for low-cost magnetometers was carried out by the authors. Seven algorithms were selected for this purpose according to their popularity and their performance. A standard, unified, and complete linear measurement model was used here as the reference model for analyzing all calibration methods. After establishing the full calibration problem, these seven algorithms were discussed and were presented in an easy-to-implement way.

In order to evaluate fairly the presented algorithms’ performance, we proposed a method for optimally generating artificial magnetometer data. This method was used for executing a plethora of Monte Carlo simulations. The evaluation metrics we focused on were the robustness, the accuracy and the efficiency of the algorithms. We designed several experiments to check the behavior of the algorithms under different values in bias, different values in non-orthogonality errors, different number of measurements and finally under various orders of variance in noise. Finally, several datasets of real magnetometer’s data, from two different, low-cost, commercial sensors were used to verify the results obtained using the artificial data.

The following summarizes our findings regarding the studied algorithms and their possible implementation. Except from the objective criteria that we established in Section 10 to evaluate and compare the presented algorithms (accuracy, robustness, computational efficiency), in Table 4 we also evaluate the algorithms in terms of simplicity. Simplicity is used as a (subjective) metric describing our personal experience developing and testing the algorithms. It is related both to the algorithmic complexity of the algorithms (which is not an inherent disadvantage) and the quality of their presentation in the original manuscripts. The algorithms are discussed in chronological order of publication.

✓ **TWOSTEP**: Extremely time efficient. Works effectively for small distortions. Has low accuracy in general. The method can be generalized to on-orbit calibration.
✓ **Crassidis et al.**: Easy to implement. Extremely time efficient. Works effectively for small to medium distortions. The method can be generalized to on-orbit calibration. It is the only algorithm that provides online update. It can be considered as a more accurate and effective version of TWOSTEP with similar time complexity.
✓ **Dorveaux et al.**: Easy to implement. Moderately time efficient. Robust and accurate, but vulnerable to large values of bias.
✓ **Vasconcelos et al.**: Difficult to implement. Characterized by high time-complexity. Exceptional accuracy and robustness for small distortions.
✓ **Ali et al.**: Robust and accurate. Very high computational cost. Some prior knowledge of the search space is beneficial. At the beginning of the algorithm, the unknown variables are randomized and, thus, it is not always ensured that the algorithm will reach an optimal point. Thus, a couple of repetitions might be needed. Using modern PSO algorithms which can constrain the search space and handle a few variable inequalities increases the algorithm’s performance significantly.
✓ **Wu and Shi**: Difficult to implement. Characterized by high time-complexity. Exceptional accuracy even with larger distortion. We noticed a 1% failure of finding an initial estimate due to inadequacy of applying Cholesky decomposition.
✓ **MAG.I.C.AL**: Easy to implement. Moderately time efficient. Exceptional robustness and accuracy in both synthetic and real data experiments.

To conclude, in this work, we tried to cover a broad range of realistic cases and test the limits of the algorithms, noting that in real life the performance requirements differ from application to another. In some applications computational efficiency may be of major importance while great accuracy may not be needed, while in others, a very accurate calibration is essential even if significantly more computation time is required for this. Thus, there is no “perfect” algorithm appropriate for all applications; different algorithms may be more appropriate for different cases. 

## Figures and Tables

**Table 1:** Notation.

∥.∥	Euclidean Norm
${∥.∥}_{F}$	Frobenius Norm
vec (·)	Vectorization of Matrix
diag(·)	Diagonal Matrix
chol (·)	Cholesky Factorization
$I_{n\times n}$	n×n Identity Matrix
$0_{m\times 1}$	m×1 Zero Vector
N	Normal Distribution
U	Uniform Distribution
∇	Gradient Vector
$\nabla^{2}$	Hessian Matrix
⊗	Kronecker Product
O(3)	Orthogonal Group of dimension 3
SO(3)	3D Rotation Group
U(3)	Group of 3×3 Upper Triangular Matrices

**Table 2:** Baseline evaluation of the presented algorithms.

Algorithm	Accuracy (1/ρ)	Robustness (RB%)	Execution Speed (1/τ)
TWOSTEP [1]	$35.3\times 10^{0}$	91.6%	455 $s^{-1}$
Crassidis et al. [6]	$3.31\times 10^{3}$	100%	47.6 $s^{-1}$
Dorveaux et al. [3]	$2.26\times 10^{5}$	100%	12.8 $s^{-1}$
Vasconcelos et al. [2]	$2.28\times 10^{5}$	99.6%	0.089 $s^{-1}$
Ali et al. [7]	$2.27\times 10^{5}$	98.8%	0.10 $s^{-1}$
Wu and Shi [4]	$2.32\times 10^{5}$	87.2%	0.24 $s^{-1}$
MAG.I.C.AL [5]	$2.28\times 10^{5}$	100%	29.4 $s^{-1}$

**Table 3:** Operation parameters of the two magnetic field sensors.

	BNO055	LSM9DS1TR
Measurement Range	±13 Gauss	±4 Gauss
Sampling Rate	30 Hz	80 Hz
Measurement Resolution	16 bits	16 bits

**Table 4:** Algorithms’ Comparison Summary – More checkmarks correspond to better performance regarding a specific metric.

Algorithm	Simplicity	Robustness	Accuracy	Efficiency
TWOSTEP	✓✓	✓✓	✓	✓✓✓
Crassidis et al.	✓✓✓	✓✓	✓	✓✓✓
Dorveaux et al.	✓✓✓	✓✓✓	✓✓✓	✓✓
Vasconcelos et al.	✓	✓	✓✓	✓
Ali et al.	✓✓	✓✓✓	✓✓✓	✓
Wu and Shi	✓	✓	✓✓✓	✓
MAG.I.C.AL	✓✓✓	✓✓✓	✓✓✓	✓✓

## Appendix A

### Appendix A.1. Gradient Vector and Hessian Matrix for the Algorithm of Vasconcelos et al.

This section (Section 6) presents the analytical algebraic expressions for the gradient and Hessian of the likelihood function (35), used in descent optimization methods. Let $u_{k}=y_{k}-h$, the gradient of the likelihood function *J* (33) is denoted by ${\nabla J|}_{x}=\left[{\nabla J|}_{\hat{T}}{\nabla J|}_{h}\right]$ and described by the submatrices

(A1a) $$\begin{array}{cc} {\nabla J|}_{\hat{T}} & =\sum_{k=0}^{N}\frac{2c_{k}}{\sigma^{2}}u_{k}⊗\hat{T}u_{k} \end{array}$$

(A1b) $$\begin{array}{cc} {\nabla J|}_{h} & =\sum_{k=0}^{N}\frac{-2c_{k}}{\sigma^{2}}{\hat{T}}^{T}\hat{T}u_{k} \end{array}$$

where $c_{k}=1-{∥\hat{T}u_{k}∥}^{-1}$. The Hessian

(A2) $$\nabla^{2}{J|}_{x}=\left[\begin{array}{cc} H_{\hat{T},\hat{T}} & H_{\hat{T},h}\\ H_{\hat{T},h}^{T} & H_{h,h} \end{array}\right]$$

is given by the following submatrices

(A3a) $$\begin{array}{cc} H_{\hat{T},\hat{T}} & =\sum_{k=1}^{N}\frac{2}{\sigma^{2}}\left[\frac{\left(u_{k}u_{k}^{T}\right)⊗\left(\hat{T}u_{k}u_{k}^{T}{\hat{T}}^{T}\right)}{{∥\hat{T}u_{k}∥}^{3}}+c_{k}\left[\left(u_{k}u_{k}^{T}\right)⊗I_{3\times 3}\right]\right] \end{array}$$

(A3b) $$\begin{array}{cc} H_{\hat{T},h} & =\sum_{k=1}^{N}\frac{-2}{\sigma^{2}}\left[\frac{\left(u_{k}⊗\hat{T}u_{k}\right)u_{k}^{T}{\hat{T}}^{T}\hat{T}}{{∥\hat{T}u_{k}∥}^{3}}+c_{k}\left[u_{k}⊗\hat{T}+I_{3\times 3}⊗\hat{T}u_{k}\right]\right] \end{array}$$

(A3c) $$\begin{array}{cc} H_{h,h} & =\sum_{k=1}^{N}\frac{2}{\sigma^{2}}\left[\frac{{\hat{T}}^{T}\hat{T}u_{k}u_{k}^{T}{\hat{T}}^{T}\hat{T}}{{∥\hat{T}u_{k}∥}^{3}}+c_{k}{\hat{T}}^{T}\hat{T}\right] \end{array}$$

### Appendix A.2. Gradient Vector and Hessian Matrix for the Algorithm of Wu and Shi

This section (Section 8) presents the algebraic expressions for the gradient and Hessian of the likelihood function (51), used in descent optimization methods. For notational simplicity $\hat{T}$ and $\hat{m}$ are replaced by *T* and *m*. Let $u_{k}=y_{k}-h$, the Jacobian vector and Hessian matrix are, respectively, derived as

(A4) $${\nabla J|}_{x}={\left[\begin{array}{cccc} J_{T}^{T} & J_{h}^{T} & \underset{k=1:N}{\underset{︸}{J_{m_{k}}^{T}}} & \underset{k=1:N}{\underset{︸}{J_{\lambda_{k}}^{T}}} \end{array}\right]}^{T}$$

(A5) $$\nabla^{2}{J|}_{x}=\left[\begin{array}{cccc} H_{TT} & H_{Th} & H_{Tm_{k}}\ldots & 0_{9\times 1}\ldots \\ H_{Th}^{T} & H_{hh} & H_{hm_{k}}\ldots & 0_{3\times 1}\ldots \\ H_{Tm_{k}}^{T} & H_{hm_{k}}^{T} & H_{m_{k}m_{k}}\ldots & H_{m_{k}\lambda_{k}}\ldots \\ \vdots & \vdots & \vdots & \vdots \\ 0_{9\times 1}^{T} & 0_{3\times 1}^{T} & H_{m_{k}\lambda_{k}}^{T}\ldots & 0\ldots \\ \vdots & \vdots & \vdots & \vdots \end{array}\right]$$

where

(A6) $$\begin{array}{cc} J_{T} & =-2\sum_{k=1}^{N}m_{k}⊗\left(u_{k}-Tm_{k}\right)\\ J_{h} & =-2\sum_{k=1}^{N}\left(u_{k}-Tm_{k}\right)\\ J_{m_{k}} & =-2T^{T}\left(u_{k}-Tm_{k}\right)+2\lambda_{k}m_{k}\\ J_{\lambda_{k}} & ={∥m_{k}∥}^{2}-1 \end{array}$$

and

(A7) $$\begin{array}{cc} H_{TT} & =2\sum_{k=1}^{N}\left(m_{k}m_{k}^{T}\right)⊗I\\ H_{Th} & =2\sum_{k=1}^{N}m_{k}⊗I\\ H_{Tm_{k}} & =2\left(\left(m_{k}⊗I\right)T-I⊗\left(u_{k}-Tm_{k}\right)\right)\\ H_{hh} & =2NI\\ H_{hm_{k}} & =2T\\ H_{m_{k}m_{k}} & =2T^{T}T+2\lambda_{k}I\\ H_{m_{k}\lambda_{k}} & =2m_{k} \end{array}$$

Note that in [4], the calibration matrix, *T*, is considered to be an upper triangular matrix. Thus, from both the gradient vector and the Hessian matrix, the rows and columns that correspond to the lower triangular elements of *T* must be removed.
